# Preventive Effect of Yuzu and Hesperidin on Left Ventricular Remodeling and Dysfunction in Rat Permanent Left Anterior Descending Coronary Artery Occlusion Model

**DOI:** 10.1371/journal.pone.0110596

**Published:** 2015-01-05

**Authors:** Hye Yon Yu, Ji Hun Ahn, Se Won Park, Yi-Sook Jung

**Affiliations:** 1 Department of Physiology, School of Medicine, Ajou University, Suwon, Republic of Korea; 2 College of Pharmacy, Ajou University, Suwon, Republic of Korea; 3 Department of Cardiology, Soon Chun Hyang University Gumi Hospital, Gumi, Republic of Korea; 4 Molecular Biotechnology, College of Life and Environmental Sciences, Konkuk University, Seoul, Republic of Korea; 5 Research Institute of Pharmaceutical Sciences and Technology, Ajou University, Suwon, Republic of Korea; Scuola Superiore Sant'Anna, Italy

## Abstract

Left ventricular (LV) remodeling, which includes ventricular dilatation and increased interstitial fibrosis after myocardial infarction (MI), is the critical process underlying the progression to heart failure. Therefore, a novel approach for preventing LV remodeling after MI is highly desirable. Yuzu is a citrus plant originating in East Asia, and has a number of cardioprotective properties such as hesperidin. However, no study has proved whether yuzu can prevent LV remodeling. The aim of this study was to determine the effects of yuzu on heart failure (HF) and its potential impact on the LV remodeling process after MI. Our *in vivo* study using the permanent left anterior descending coronary artery (LAD) occlusion model demonstrate that one week pre-treatment with yuzu or its major metabolite hesperidin before LAD occlusion significantly attenuated cardiac dysfunction, myocyte apoptosis and inflammation. Not only yuzu but also hesperidin inhibited caspase-3 activity, myeloperoxidase expression, α-smooth muscle actin expression, and matrix metalloproteinase-2 activity in a permanent LAD occlusion rat model. To our knowledge, our findings provide the first evidence that yuzu and hesperidin prevent MI-induced ventricular dysfunction and structural remodeling of myocardium.

## Introduction

LV remodeling is pathologic changes in the architecture of the LV that occur due to various cardiovascular diseases (CVDs) including MI and hypertension [1]. Of these, MI is caused by the partial interruption or occlusion of the blood supply to a part of the myocardium. This most commonly involves the occlusion of a coronary artery following the rupture of a vulnerable atherosclerotic plaque [2]. LV remodeling after MI is associated with a combination of pathologic conditions, including myocyte hypertrophy, myocyte apoptosis, myofibroblast proliferation, inflammatory reaction, and interstitial fibrosis, which ultimately lead to the loss of systolic and diastolic function [3]. Cardiac hypertrophy is a compensatory process in response to increased hemodynamic overload, characterized by an increase in the size of individual cardiac myocytes and wall thickness. On the other hand, in chronic MI following LAD occlusion, a transition occurs from compensatory cardiac hypertrophy to decompensatory hypertrophy, characterized by a chamber dilation and wall thinning. In this chronic condition, processes such as extracellular matrix turnover, fibrosis, inflammation and apoptosis are crucial determinants [4], [5].

LV remodeling after MI is a key contributor to HF, which is one of the most common causes of cardiovascular morbidity and mortality worldwide [6]. Conventional HF therapy is still largely based on targeting the causes and neurohumoral activation of HF, and includes agents such as angiotensin-converting enzyme inhibitors, angiotensin-receptor antagonists, beta-blockers, and aldosterone antagonist [7]. Recently, natural products have become popular worldwide and have gained wide acceptance as adjuncts to conventional therapy. Various studies have shown natural products such as grapes, citrus fruits, broccoli, and cacao are rich sources of phytochemicals such as polyphenols that are well known for their antioxidant and cardioprotective effects [8], [9], [10]. Furthermore, epidemiological evidence indicates that a negative correlation exists between the consumption of flavonoid-rich foods and the incidence of CVDs [11].

Yuzu (*Citrus junos* Sieb ex Tanaka), one of the natural products receiving attention for their health benefits, is a citrus fruit native to northeast Asia, including Korea, China, and Japan. It has been used in traditional medicine in northeast Asia, and it is known to improve blood circulation and prevent colds [12]. We have previously reported that yuzu and its major compounds inhibit platelet aggregation *in vivo* and *in vitro* [12]. However, little report has described whether yuzu has beneficial effect against cardiac dysfunction following chronic MI. The goal of the present study was to evaluate the effects of yuzu in a rat model of LV remodeling induced by permanent LAD occlusion. Considering that hesperidin is well-known major functional component of yuzu, we have also evaluated whether hesperidin contributes to the protective effect of yuzu.

## Materials and Methods

### Materials

Ethanolic extract of yuzu were obtained from Konkuk University (Seoul, Republic of Korea). Briefly, Yuzu fruits minced were extracted with ethanol and lyophilized to remove solvent. Yuzu extract was dissolved in saline (0.9% NaCl) for the *in vivo* study. Hesperidin and polyethylene glycol (PEG) were purchased from Sigma Chemical Co. (St. Louis, MO, USA). Because hesperidin is water-insoluble, it was dissolved in 70% PEG which is a widely used solvent for water-insoluble compounds in *in vivo* study [13].

### Experimental protocol

All experimental procedures conformed to the Guide for the Care and Use of Laboratory Animals published by the US National Institutes of Health (NIH Publication No. 85-23, revised 1996), and the Committee on Animal Research at Ajou Medical Center, Ajou University (Suwon, Republic of Korea), approved the study. Male Sprague-Dawley rats (weight, 250–300 g) were anaesthetized with an intraperitoneal injection of ketamine (100 mg/kg) and xylazine (10 mg/kg) before surgery. The body temperature of the rats was maintained at 37±0.5°C during surgery by using a thermostatically controlled warming plate as described previously [14]. In the loss-of-function study, ischaemia-induced myocardial injury was induced by ligating the LAD artery as described previously [9], [14]. Sham-operated control group (sham) underwent the same surgical procedures except that the suture placed under the left anterior descending was not tied. At 4 weeks after LAD occlusion, rats were euthanized by CO_2_ inhalation for heart isolation. LV was used for staining experiments. Infarct and peri-infarct zone of left ventricle were used for gelatin zymography and western blot analysis. Peri-infarct zone was defined as the area within 2 mm of the visible edge of infarction [15].

**Figure 1:**
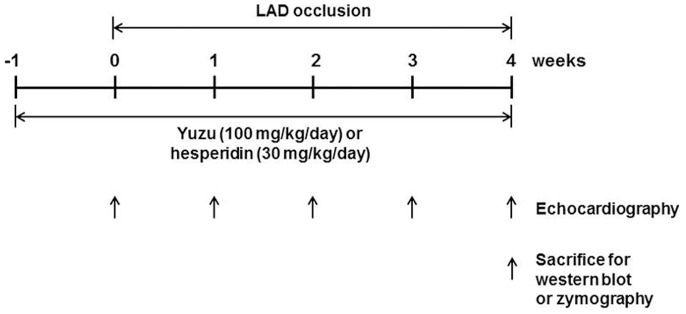
Experimental schedule.

The rats were randomly distributed into experimental pre-treatment groups with similar body weights. Yuzu (100 mg/kg/day, n = 20), hesperidin (30 mg/kg/day, n = 20), vehicle (PEG 0.3 ml/day, n = 23) or sham (PEG 0.3 ml/day, n = 20) was administered by oral gavage once daily beginning 7 days before LAD occlusion and continuously administered until the time of the terminal study, using oral gavage around 10∶00 A.M. every morning (for total 5 weeks, **Figure 1**).

### Echocardiography

The rats were subjected to transthoracic echocardiography. In brief, the rats were anaesthetized with an intraperitoneal injection of ketamine (100 mg/kg) and xylazine (10 mg/kg) and were examined with non-invasive echocardiography (echocardiograph IE33 ultrasound, S12-4 probe, Philips). Ventricular remodeling in the vehicle-, yuzu-, or hesperidin-treated groups was assessed weekly with serial echocardiography (beginning 0 week prior to LAD occlusion until 4 weeks after LAD occlusion). Cardiac ventricular dimensions were measured on M-mode images (left parasternal long axis). And the average of 5 consecutive cardiac cycles of each animal is reported.

### Histological analysis

LV tissue was fixed with 4% paraformaldehyde for 24 hours, dehydrated with increasing concentrations of ethanol, and then embedded in paraffin. LV sections (5 µm) were stained with hematoxylin and eosin (H&E, Sigma-Aldrich) or Masson trichrome (polysciences) as previously described [8], [16], [17]. Images were captured by utilizing a Zeiss Axioplot Vision-series microscope and software (Carl Zeiss, Oberkochen, Germany) and were quantified using the NIH Image J analysis program (NIH, Bethesda, MD, USA).

### Immunohistochemistry

Immunostaining was performed using a streptavidin–biotin-immunoperoxidase complex method with 5 µm thick sections, which had been deparaffinized and heated in 0.01 M citrate buffer solution (pH ¼6.0) for 15 min for antigen retrieval. Rabbit polyclonal antibody against, myeloperoxidase (MPO), or cardiac tronponin I (cTnI) purchased from Abcam (Cambridge, UK) was used. The slides were examined using a light microscope (Olympus CX21, Japan) and was evaluated with reference to the optical density of the stain by using a computer-assisted image analysis system, ImageJ1.45F (NIH, USA).

### Gelatin zymography

Gelatin zymography was performed utilizing the Novex In-gel Zymography System (Invitrogen, Carlsbad, CA, USA), according to the manufacturer’s instructions. Heart tissues were homogenized in 50 mM Tris-HCl (pH 7.5) containing 150 mM NaCl and 5 mM CaCl_2_. After centrifugation, the supernatants were harvested and 25 µg of protein was mixed with Tris-glycine sodium dodecyl sulfate (SDS) sample buffer. The samples were run on a Novex 10% Zymogram Gelatin Gel, followed by incubation with Zymogram Renaturing Buffer and subsequent incubation with Zymogram Developing Buffer. After an overnight reaction, the gel was stained with Simply Blue Safe Stain (Invitrogen).

### Western blot analysis

Heart tissues were homogenized in a buffer containing 50 mmol/L Tris-HCl pH 7.4, 1% NP-40, 150 mmol/L NaCl, 0.25% Na-deoxycholate, 2 mmol/L EDTA, 1 mmol/L NaF, 1 mmol/L Na_3_VO_4_, 1 mmol/L PMSF, 10 µg/mL aprotinin, and 10 µmol/L leupeptin. Homogenates were centrifuged at 14, 681 *g* for 15 min and the supernatants were collected as previously described [18]. Equal amounts of protein were then separated by SDS-polyacrylamide gel electrophoresis (PAGE) and reacted with antibodies specific for caspase-3 (Cell Signaling, Danvers, MA, USA), α-SMA (Abcam) and α-tubulin (Sigma-Aldrich). After probing with an HRP-conjugated secondary antibody, the proteins were visualized using LAS 1000 (Fuji Photo Film, Tokyo, Japan). Densitometric analyses were performed using Quantity One software, ImageJ1.45F (NIH, USA).

### Terminal dUTP nick end-labeling (TUNEL) staining

In situ labeling of fragmented DNA was performed using the Apop Taq Plus Kit (Oncor, Gaithersburg, MD). Briefly, nucleosome-sized DNA fragments were labeled with digoxigenin nucleotide and reacted with peroxidase-conjugated anti-digoxigenin antibody and 3, 3′-diaminobenzidine as previously described [14]. The percent cell death was calculated by expressing the number of TUNEL-positive cells as a percentage of total cell counts.

### Statistical analysis

All data are expressed as means ± SDs. The results were analyzed using 2-way ANOVA and the differences between groups were compared by using the Student’s t-test. A p value of <0.05 was considered statistically significant. All experiments were repeated at least 4 times.

## Results

### Yuzu and hesperidin prevented LV remodeling and functional deterioration following MI

**Figure 2:**
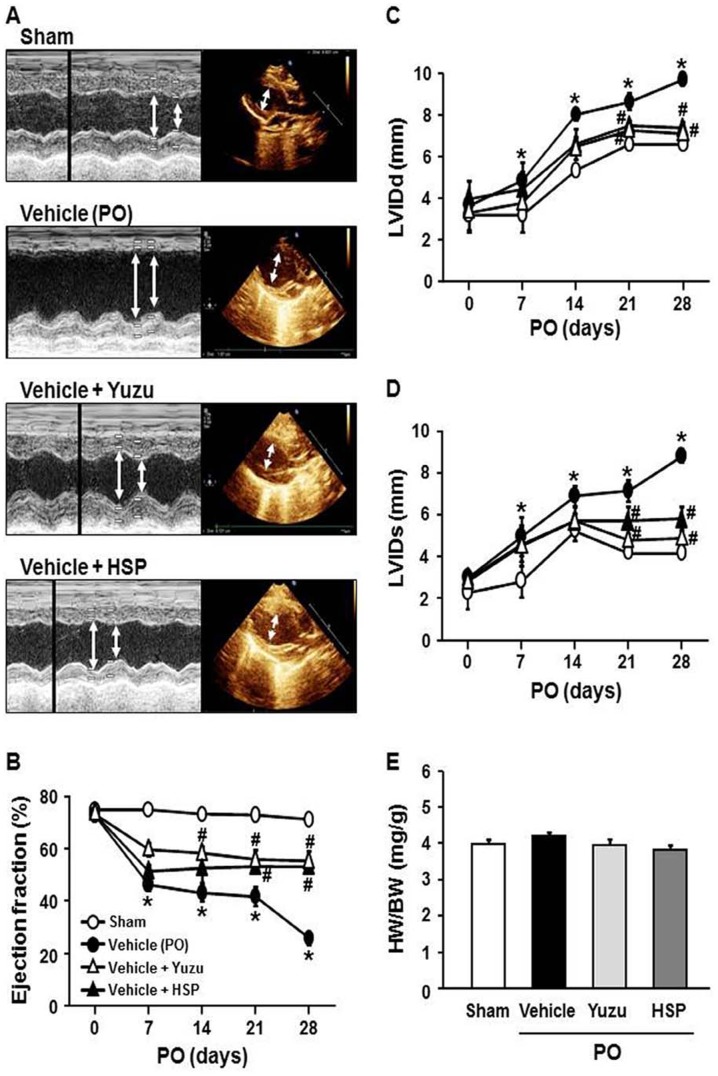
Effect of pre-treatment with yuzu (100 mg/kg/day) or hesperidin (HSP, 30 mg/kg/day) on cardiac dysfunction. (A) Representative M-mode echocardiograms. (B–E) Echocardiographic measurement of left ventricular (LV) internal dimensions at both systole and diastole (LVIDs and LVIDd, respectively), LV ejection fraction, and heart weight (HW) to body weight (BW) ratio. Data are expressed as mean ± SEM; *p<0.05 vs. sham; #p<0.05 vs. vehicle (permanent occlusion, PO). n = 10–13.

In this study, we evaluated the effects of yuzu and hesperidin on LV remodeling in a rat model of permanent LAD occlusion. The vehicle-treated group showed a significantly greater increase in end-diastolic and end-systolic dimensions (LVIDd & LVIDs) compared to sham group (**Figure 2A–D**). This was associated with marked LV dysfunction as reflected by reduced LV ejection fraction (EF). LV function was preserved more markedly in the yuzu- or hesperidin-treated groups up to the termination of the study. However, cardiac hypertrophy as measured by the HW/BW ratio no differences among the groups at the 4 weeks after LAD occlusion (**Figure 2E**).

### Yuzu and hesperidin prevented myocardial fibrosis during LV remodeling after chronic MI

**Figure 3:**
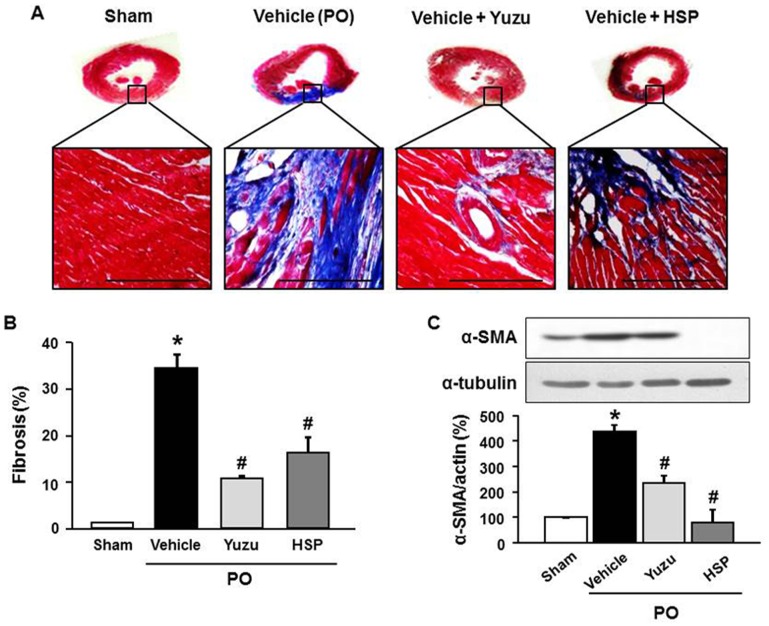
Effect of pre-treatment with yuzu (100 mg/kg/day) or hesperidin (HSP, 30 mg/kg/day) on cardiac fibrosis. (A) Representative Masson’s trichrome sections of the left ventricle. Scale bar, 100 µm. n = 6–7. (B) Quantitative analysis of fibrosis. Data are expressed as mean ± SEM; *p<0.05 vs. sham; #p<0.05 vs. vehicle. n = 6–7. (C) Expression of myocardial α-smooth muscle actin (α-SMA) protein. Representative image (upper part) and quantitative analysis (bottom part). Scale bar, 100 µm. n = 7–8. PO, permanent occlusion.

To determine whether our *in vivo* findings have pathological relevance, we evaluated the effects of yuzu and hesperidin on cardiac fibrosis in a rat MI model of permanent LAD occlusion. The rats were treated with yuzu, hesperidin, or vehicle 1 week before LAD occlusion. As observed upon Masson’s trichrome staining, increased interstitial fibrosis was observed in the rats treated with the vehicle after 4 weeks of LAD occlusion, which was significantly reduced in the group that was pre-treated with yuzu or hesperidin (**Figure 3A and B**). Western blot analysis revealed upregulation of α-SMA expression in hearts from the vehicle group, which was reversed to control levels by pre-treatment with yuzu or hesperidin (**Figure 3C**).

### Yuzu and hesperidin reduced the inflammatory reaction and MPO expression during LV remodeling after chronic MI

**Figure 4:**
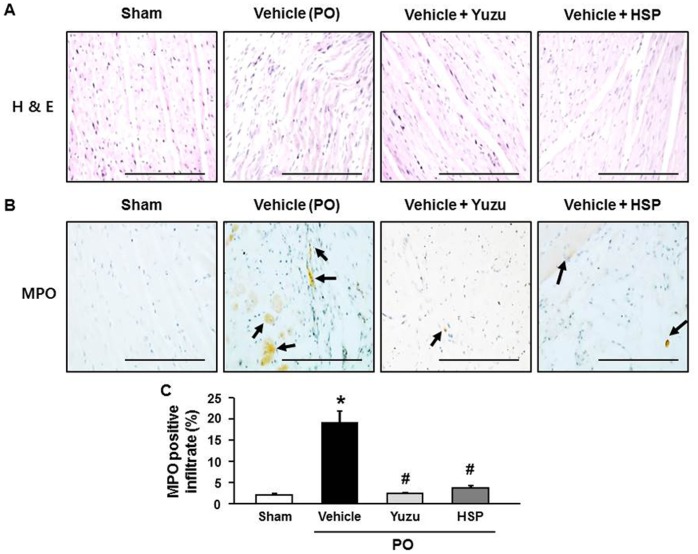
Effect of pre-treatment with yuzu (100 mg/kg/day) or hesperidin (HSP, 30 mg/kg/day) on inflammatory reaction. (A) Representative H & E staining sections of the left ventricle (LV). Scale bar, 100 µm. n = 6–7. (B) Representative examples of neutrophil activity expressed as myeloperoxidase (MPO) antibody activity (arrow) in the LV. Scale bar, 100 µm. n = 6–7. (C) Quantitative analysis of MPO positive cells. Data are expressed as mean ± SEM; *p<0.05 vs. sham; #p<0.05 vs. vehicle. n = 6–7.

To histologically confirm that myocardial injury reflected inflammation, we performed H&E staining and MPO immunohistochemical analysis to detect neutrophil activity. H&E staining showed that the strongest inflammatory reactions were observed in the vehicle group. Examination of heart sections of the yuzu- and hesperidin-treated groups showed nearly normal cardiac cells with a well-preserved cytoplasm and prominent nucleolus (**Figure 4A**). The number of MPO-expressing cells was significantly higher in the vehicle groups than in the yuzu- and hesperidin-treated groups (**Figure 4B and C**).

### Yuzu and hesperidin block myocardial apoptosis and LV wall thinning through inhibition of MMP-2 activation during LV remodeling after chronic MI

**Figure 5:**
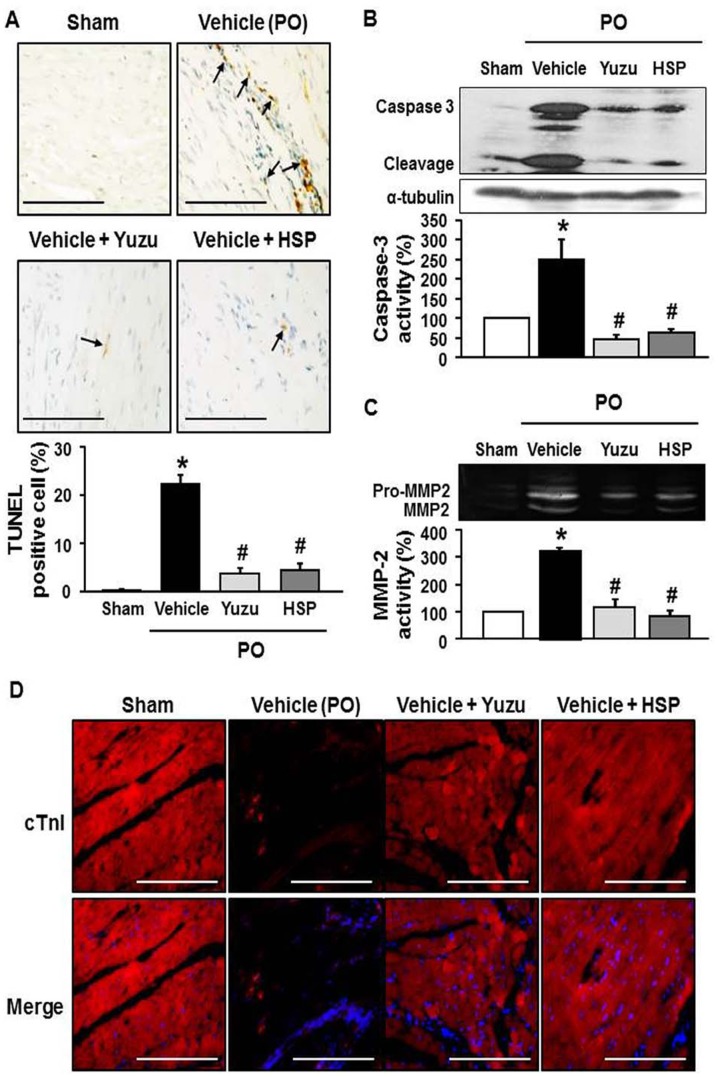
Effect of pre-treatment with yuzu (100 mg/kg/day) or hesperidin (HSP, 30 mg/kg/day) on cardiomyocyte apoptosis. (A) Representative TUNEL staining (upper images) and quantitative analysis of TUNEL positive cells (bottom graph). Data are expressed as mean ± SEM; *p<0.05 vs. sham; #p<0.05 vs. vehicle. Scale bar, 100 µm. n = 6–7. (B) The activity of myocardial caspase-3 protein. Representative image (upper part) and quantitative analysis (bottom part). n = 7–8. (C) The activity of MMP-2. Representative image (upper part) and quantitative analysis (bottom part). n = 7–8. (D) Representative cardiac Troponin I (cTnI) staining. Scale bar, 100 µm. n = 6–7. PO, permanent occlusion.

We next investigated whether adverse LV remodeling contributed to increased cardiac cell death in the rats with LV remodeling after MI. Apoptosis was evaluated using the TUNEL assay. After 4 weeks of LAD occlusion, the number of TUNEL-positive myocytes was significantly higher in the vehicle groups than in the yuzu- and hesperidin-treated groups (**Figure 5A**). Thus, yuzu and hesperidin attenuated MI-induced myocardial apoptosis. Next, we compared the caspase-3 activity of the yuzu- and hesperidin-treated groups with the vehicle group to determine whether caspase-3 is involved in myocardial apoptosis. Treatment with yuzu or hesperidin significantly reduced caspase-3 activity to levels similar to those observed in the sham-operated animals (**Figure 5B**).

To gain a better understanding of the mechanisms leading to the prevention of LV wall thinning in yuzu- and hesperidin-treated groups, we examined MMP-2 activity and resident cardiomyocytes in the LV myocardium. MMP-2 activity increased in the left ventricles of rats subjected to LAD occlusion, whereas treatment with yuzu significantly reduced MMP-2 activity to levels similar to those observed in sham-operated animals (**Figure 5C**). LAD occlusion for 4 weeks induced prominent cardiomyocyte loss in the LV mass, as demonstrated by decreased cTnI staining. Compared to vehicle treatment, yuzu or hesperidin treatment significantly inhibited cardiomyocyte loss and expression of cTnI (**Figure 5D**). These effects were associated with a significant improvement in ventricular remodeling and dysfunction.

## Discussion

Herein, we provide the first evidence for the cardioprotective role of the ethanolic extract of yuzu and its major component, hesperidin in rat chronic MI model induced by permanent LAD occlusion, by demonstrating that the ethanolic extract of yuzu and hesperidin prevented heart from MI-induced dysfunction, LV fibrosis, inflammatory reaction and myocyte apoptosis.

Chronic MI results in complex architectural alterations such as dilatation of the LV and infarct thinning, which is called LV remodeling, in both infarct and non-infarct region. Patients exhibiting extensive LV remodeling after MI are more likely to experience complications such as HF and myocardial rupture, leading to an elevated risk of mortality [19]. Although modern cardiology has made substantial advances in the diagnosis and management of MI, it is necessary to design therapeutic strategies to attenuate LV remodeling after MI by modulation of the molecular and cellular factors involved in the multiple components of LV remodeling [20].

After chronic MI, LV fibrosis contributes to adverse structural remodeling, leading to impaired contractile properties of the LV as well as deteriorated electrical conduction system [21]. LV fibrosis occurs as a result of the imbalance between enhanced synthesis reduced degradation of collagen [6]. As a critical step in response to myocardial injury, fibroblasts are activated into α-SMA-positive myofibroblasts which can generate extracellular matrix proteins such as type I collagen. Therefore, the degree of fibroblast activation is a significant predictor of HF progression in both experimental animal models and in human patients [22]. In the present study, we have demonstrated that the ethanolic extract of yuzu and hesperidin exert anti-fibrotic remodeling effects via the inhibition of excess collagen deposition and conversion of fibroblasts into α-SMA-positive myofibroblasts during LV remodeling process following permanent LAD occlusion. These results suggest that yuzu and hesperidin represent novel preventive natural products against LV remodeling and cardiac dysfunction induced by chronic MI.

Regarding cardiac structure remodeling process after chronic MI, recent studies have identified the importance of several inflammatory mediators that are released during this process, such as neutrophils and various cytokines, and inflammatory cells are attracted to the myocardial injury site [23], [24], [25]. MPO is a well-known enzyme that is released by activated neutrophils and has powerful pro-oxidative and pro-inflammatory properties [26], [27]. Recent studies have suggested that regional MPO activity may contribute to right ventricular failure and ischaemic-perfusion injury [23], [26]. Subsequent studies have provided quantitative support for this observation by showing significant elevations in the systemic levels of MPO in a wide spectrum of CVD scenarios, with chronic MI and HF being the most frequently studied [27]. Accordingly, pre-clinical studies in experimental models suggest a possible therapeutic role of MPO inhibition in HF. In this study, we found that yuzu and hesperidin have anti-inflammatory properties and participate in the control of the inflammatory response through the inhibition of MPO expression during chronic MI.

Experimental models and clinical studies have shown that loss of functional cardiomyocytes contributes to structural changes that underlie progressive LV remodeling during chronic MI [6], [28]. Although the significance of apoptosis in LV remodeling still remains debatable, cardiomyocyte apoptosis leads to the loss of cardiomyocyte mass and the reduction of myocardial contractile function [29]. Therefore, elimination of pro-apoptotic signals may prevent the progression of LV remodeling during chronic MI. Since the main apoptotic death pathways converge on caspases, the most efficient approach for interrupting cardiomyocyte apoptosis might be the targeting of these enzymes. Many animal studies have confirmed that the inhibition of caspases, such as caspase-3, mitigates LV dysfunction and enables survival during the progression to end-stage HF. Although specific caspase inhibitors are being developed and a few have shown promising results for clinical therapy by targeting cell death in LV remodeling, no clinical trials have been performed to inhibit apoptotic pathways during MI until very recently [30]. In the present study, we have demonstrated that yuzu and hesperidin have anti-apoptotic properties and participate in the control of cell death through inhibition of caspase-3 activity and expression.

Cardiomyocyte loss during ischemic damage can be replaced by non-contractile fibrotic cells rather than by new cardiomyocytes [31]. In humans, it is known that extensive LV dilatation after chronic MI increases the risk of complications such as the HF, aneurysm formation and cardiac rupture. In addition, the positive effects of MMP inhibition on LV dilatation in animal models have led to the proposed use of MMP inhibitors as potential therapies in patients at risk for the development of HF after MI [19]. MMP-2 is abundant and ubiquitously expressed in almost all of the cells that comprise the heart. Activated MMP-2 degrades susceptible sarcomeric and cytoskeletal proteins including troponin I (TnI), myosin light chain-1 (MLC-1), and α-actin, leading to the acute contractile dysfunction observed in ischemia/reperfusion injury [32]. Interestingly, the present study have demonstrated that yuzu and hesperidin effectively preserved cardiomyocyte mass during chronic MI, possibly through the inhibition of MMP-2-induced wall thinning.

LV remodeling after chronic MI remains a major cause of morbidity and mortality worldwide, leading to a dramatical increase of health care costs [33]. Despite a number of pharmacological advances, mortality following MI remains still high. The present study has demonstrated that pre-treatment of the ethanolic extract of yuzu or hesperidin significantly prevented LV remodeling process LV dysfunction following chronic MI, and suggested a potential use of yuzu or hesperidin as a cardioprotective strategy.
